# Experiences of co-producing person-centred and cohesive clinical pathways in the national system for knowledge-based management in Swedish healthcare: a qualitative study

**DOI:** 10.1186/s40900-024-00565-3

**Published:** 2024-06-07

**Authors:** Sylvia Määttä, Christina Petersson, Boel Andersson Gäre, Göran Henriks, Henrik Ånfors, Christin Lundberg, Ylva Nilsagård

**Affiliations:** 1 Institute of Health and Care Sciences, Sahlgrenska Academy, Gothenburg University, Gothenburg, Sweden; 2 Jönköping Academy for Improvement of Health and Welfare, Jönköping University, Center for Learning and Innovation at Region Jönköping County, Sweden, Sweden; 3 Jönköping Academy for Improvement of Health and Welfare, Jönköping University, Sweden and Futurum, Region Jönköping County, Jönköping, Sweden; 4 Yerevan State University, Strategic Advisor Region Jönköping County, Yerevan, Sweden; 5 Qulturum – Center for Learning and Innovation, Region Jönköping County, Jönköping, Sweden; 6 Region Västra Götaland, Gothenburg, Sweden; 7 University Healthcare Research Centre, Faculty of Medicine and Health, Örebro University, Örebro, Sweden

**Keywords:** Co-production, Experiences, Patient participation, Healthcare, Healthcare system, Management, Clinical pathways, Macro level

## Abstract

**Background:**

When the 21 Swedish county councils decided to collaborate in the creation of a national system for knowledge-based management, patient participation was mandatory. Patient and next-of-kin representatives (PR) co-produced person-centred and cohesive clinical pathways together with healthcare professionals (HPR). Research on co-production in healthcare at the national level is scarce. The aim of this study is to explore experiences of patient participation from the perspectives of both PRs and HPRs when co-producing clinical pathways within the Swedish nationwide healthcare system for knowledge-based management.

**Methods:**

A qualitative study was conducted. A strategic sample of nine PRs and eight HPRs were interviewed individually between August 2022 and January 2023 using a semi-structured interview guide. We analysed data using an inductive content analysis.

**Results:**

Three main categories were identified: (1) Finding appropriate patient representativeness; (2) Working methods that facilitate a patient perspective; and (3) Influence of the patient perspective in the clinical pathways.

**Conclusions:**

The study demonstrates the importance of patient and next-of-kin participation in the construction of clinical pathways at the national level. The results provide a platform for further research on patient participation on the national level and add to studies on if and how patient participation on this level has an impact on how the clinical pathways are put into practice at the micro level, and the support provided at the meso level. The study contributes to the growing body of literature studying patient participation and co-production.

**Trial registration:**

Region Örebro County ID 276,940. An advisory opinion was obtained from the Swedish Ethical Review Authority (2021-05899-01).

**Supplementary information:**

The online version contains supplementary material available at 10.1186/s40900-024-00565-3.

## Abstract (de)

Co-production of guidelines and clinical pathways with patients and next-of-kin representatives is increasingly emphasised at different levels in healthcare internationally. However, little has been documented and explored regarding experiences of patient participation on a national level. Patient participation was mandatory when a national system for knowledge-based management of healthcare was launched in Sweden. Knowledge management of healthcare is the collection of methods relating to creating, sharing, using and managing the knowledge and information of an organization. All 21 of Sweden’s regions and the government have supported this collaborative system consisting of multi-professional national working groups with patient representatives. The groups develop clinical pathways aiming to enhance a coordinated, equal and effective healthcare. The person-centred clinical pathways describe assessment, diagnosis, planning and evaluation for a condition, for example for patients with a hip fracture, or congestive heart failure. The study focuses on experiences of patient participation in the national working groups. We interviewed nine patient representatives and eight healthcare professional representatives from eleven different groups. Our findings show the importance of identifying and finding appropriate patient representativeness, having working methods that facilitate a patient perspective and the patient perspective influencing the clinical pathways. This study on patient participation in the construction of clinical pathways at the national level contributes to the growing body of literature on co-production of knowledge support. The findings highlight important learning for the continued development of meaningful patient participation on the national level. It also raises curiosity on how the national approach with co-production influences local levels where the guidelines are used.

## Background

Increasingly, co-production between patients, their next-of-kin and healthcare professionals is emphasised to achieve safer and more person-centred health, healthcare and health science [1–4]. Co-production is defined by Batalden [5] as “the interdependent work of users and professionals who are creating, designing, producing, delivering, assessing, and evaluating the relationships and actions that contribute to the health of individuals and populations”. Co-production has many applications [6] and is here used as an umbrella term, covering a range of “co”-words, for example co-design and co-creation between patients and healthcare professionals (for a discussion on concepts used, see e.g. [5, 7, 8]). .

There is a lack of consensus on the relations between the terms used to describe patient participation in co-production. Terms such as patient involvement, patient engagement and shared decision-making can be discerned, among others [9–12]. A suggested linking of the terms was presented by [13] Hedberg et al., which can be further discussed. In addition, the level on which patient participation takes place is often blurred. Inspired by Arnstein’s classical description of a ladder of citizen participation [14], Carman et al. [15] designed a model for patient participation at micro, meso and macro levels that often is used in the context of patient participation [9, 16]. Care meetings between patients and healthcare professionals occur at the micro level. At the meso level, patients and their next-of-kin may participate in teams working with quality improvement of healthcare services. At the macro level – hereafter termed the national level – patients can participate in co-producing national care programmes, guidelines, and clinical pathways [17].

In this article the concept of “patient participation” is used, referring to the participation of a person with lived experiences of a health issue or condition, and the term “patient representative” (PR) is used for patients representing others with experiences of the same condition or who are next-of-kin to a person with a certain condition [18].

### Patient participation at the swedish national level

Although Swedish healthcare has a high standard, there is room for improvement. Despite primarily being funded by general taxation, there are examples where Swedish healthcare does not succeed in providing equal care and equal access to healthcare services for the whole population. This challenge of inequity was one of the drivers for the development of the knowledge-based management system. The responsibility for legislation, monitoring, education, and training of healthcare professionals lies at the national level. The responsibility for both specialised healthcare and primary healthcare services rests on the 21 regional councils, while the 290 municipalities are responsible for long-term care for older people and persons with disabilities. The regional councils are combined into six larger healthcare regions to enhance an efficient use of resources through cooperation.

**Fig. 1:**
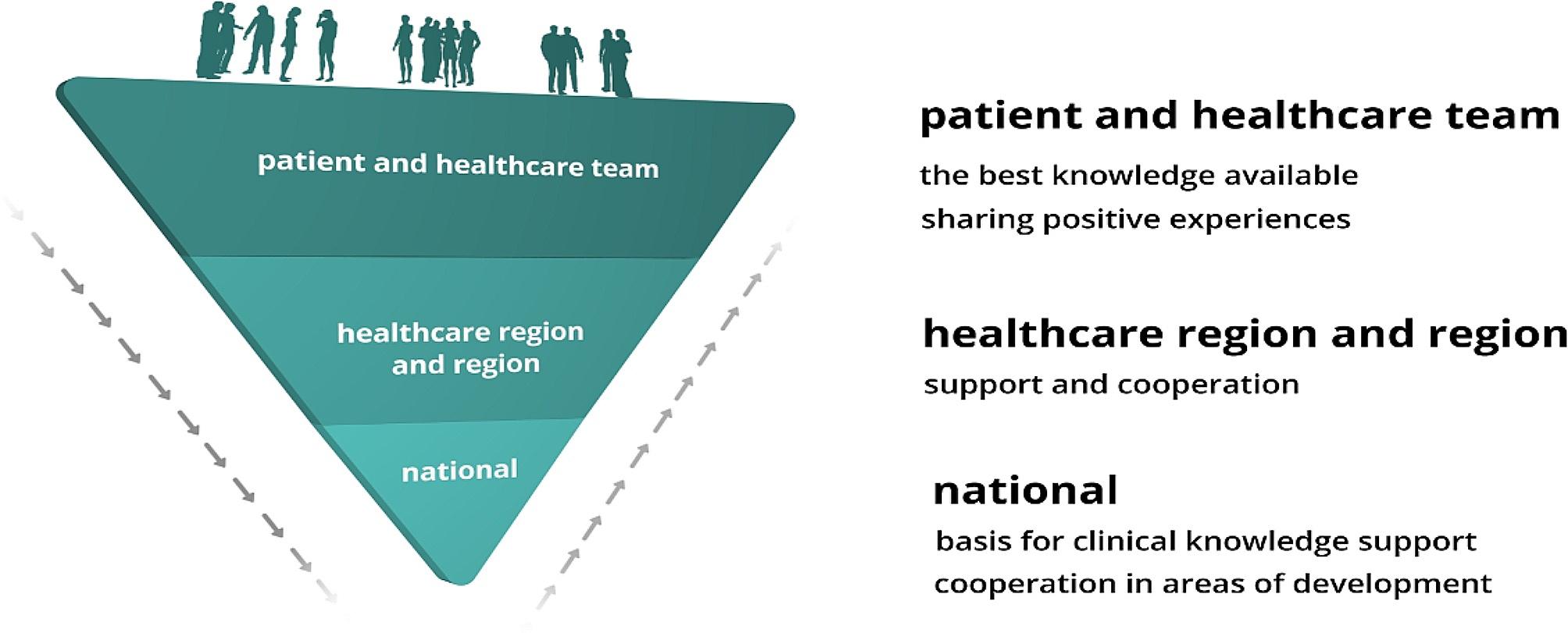
The interplay between micro (team), meso and macro system levels in the Swedish national system for knowledge-based management (figure printed with permission from SALAR’s administrative function)

In 2017, the regional councils received a recommendation to sign an agreement for a cohesive system for knowledge-based management [19]. The system was inspired by the Intermountain Healthcare system (the largest nonprofit health system in the Intermountain West, United States) [20] and formulated in accordance with Swedish healthcare laws [21]. The shared vision is: “We count our success in lives and equal health and make each other successful”. The system includes interplaying micro, meso and macro levels and is based on the interaction between knowledge support and support for follow-up, open comparison, analysis, leadership, and development (see Fig. 1). SALAR is an employer organisation and an organisation that represents and advocates local government in Sweden. All of Sweden’s municipalities and healthcare regions own and are members of SALAR.

Patient participation has from the start been considered a vital part of the system. At the national level, patient representatives and healthcare professional representatives participate in national working groups to co-produce person-centred and cohesive clinical pathways. The clinical pathways portrayed here, which will be described more later, focus on major diagnoses, intending to meet the challenges that Swedish healthcare is facing and to support a nationwide person-centred, cohesive, effective, and equal healthcare.

### Knowledge gap

The impact of patient participation in research and quality improvement projects at the micro and meso levels are explored to some extent [22–26]. However, healthcare professionals and patient representatives jointly constructing clinical pathways is a significant but less studied strategy. To our knowledge, empirical research that addresses how patient participation is experienced by patient representatives and healthcare professional representatives on a national level is scarce. It is important to study since experiences of patient participation at this level may influence future national, regional, and local initiatives. Consequently, the aim of this study is to explore experiences of patient participation from the perspectives of both patient and healthcare professional representatives when co-producing clinical pathways. The context is within the nationwide system for knowledge-based management of Swedish healthcare and if participants expressed that their participation influenced the end-products.

## Method

### Study design

We conducted a qualitative study, using an inductive content analysis following Elo and Kyngäs [27].

### Study context

The working groups in the national system of knowledge-based management constitute the context of this study. In addition to being maintained by the regions, the system has been supported by the Swedish government with a total of 90 million euros. Hosted by the six larger healthcare regions, 26 national programme groups have been established, addressing for example infectious diseases, diseases of the nervous system, and lung- and allergy diseases. Each programme group identifies conditions that could benefit from a clinical pathway and recommends starting a national multi-professional and multi-disciplinary working group that they will supervise.

A national working group produces national clinical pathways aiming to be implemented at the micro level. A clinical pathway describes assessment, diagnosis, planning, and evaluation for a specific condition. A description of the pathway from the patient perspective is mandatory. After going through an open referral process, the clinical pathways are approved for implementation throughout Swedish healthcare by the steering committee of the national system. They are presented at open digital seminars and published on a website. The meso level supports both the national and the local (micro) level as an intermediary [28]. It is the responsibility of each regional council to decide when and how the clinical pathways are put to action locally.

The larger healthcare regions, municipalities and professional organisations are invited by the national programme groups to nominate healthcare personnel to the national working groups. The nomination procedure includes a specific requirement of formal competences and disclosing conflicts of interest. Patient representatives are also recruited to the groups according to the policy and guideline for patient participation that was launched in the development of the system at an early stage, stressing that there should be at least one patient representative in each group, preferably from a national patient organisation [19]. It also states the level of remuneration to the participating patient representatives. At present, there is no formal request for a specific profile or competence of these representatives. The national programme group finally determines the composition of the working group and appoints both patient representatives and other members of the group. The national working groups are led by a chairperson and a process leader.

During the process of producing a clinical pathway, support is provided by the chairperson, the process leader, and the national administrative function for the system at SALAR (see Table 1).

**Table 1:** Examples of activities supporting members of national working groups

Patient representatives	Healthcare professional representatives
Meetings with chairperson and process leader	Meetings with process leaders and chairpersons
Exchange of experiences with other patient representatives; meetings arranged by the national support administration	Meetings with the national administrative support function
Documents (policies, routines, agreement forms etc.)	Documents (policies, routines, templates), editing services, publication on websites etc.

### Participant selection for the study

Strategic sampling was used in the study [29]. To give a broad picture of patient participation, eligible informants needed to have experience of participating in national working groups producing clinical pathways for (1) acute or chronic conditions, as well as groups that had been (2) working together for a shorter or longer period. Patient representatives, chairpersons and process leaders in the national working groups were invited to participate in the study. Eligible informants were approached and invited by email. In the email, the aim of the study was explained and, if informants were interested in being interviewed, a time for a digital interview was suggested. Informants answered by email and a digital link was sent. Informed consent was required.

The informants were encouraged to find an undisturbed, quiet place of their choice when participating in the interview. We invited nine patient representatives and nine healthcare professional representatives, of which one declined participation due to lack of time.

The final sample thus consisted of 17 informants: nine patient representatives (3 males) and eight healthcare professional representatives (3 males) (two chairpersons and six process leaders). As described above, strategic sampling was used to give a broad picture of the experiences of patient participation. During the study period several national working groups (with approximately 12–17 participants in each working group) were on-going, producing clinical pathways. 3 of the 6 clinical pathways that were finalised in 2020, 4 in the 6 that were finalised in 2021 and 5 in the 12 that were finalised in 2022 were represented in the study. Informants represented 18 different national working groups, related to 11 national programme groups working with a variety of acute and chronic diseases.

### Data collection

All interviews were undertaken in an online video session, conducted in Swedish and guided by a semi-structured interview guide with open questions followed by explanatory questions to gain a deeper understanding; see additional files for the interview guide for patient (Appendix 1) and healthcare professional representatives (Appendix 2). The interview guide was iteratively discussed and refined in the research group, including two patient representatives who were not part of the interviews that followed.

Informants were encouraged to speak openly and share their perceptions and experiences. To deepen the dialogue, probing questions were posed: “Could you elaborate on that?” and “Can you give an example?”. The interviews were audio-visually recorded and transcribed verbatim. All interviews were undertaken between August 2022 and January 2023. The duration of the interviews was approximately 30 to 45 min.

### Data analysis

Data were analysed using inductive qualitative content analysis, moving from the specific to the general to develop categories that describe the phenomenon [27]. The transcribed interviews were analysed by going back and forth in the transcriptions. An individual code was given to each informant to be able to identify their specific role: patient or next-of-kin representative, or healthcare professional representative.

The analysis began with a first round of the authors (SM, CP and YN) listening to all audio-recorded interviews and then reading all transcripts to gain a sense of the whole. In this preparatory phase, units of analysis were selected. A unit of analysis was determined to be one or more sentences, or part of a sentence, that described something related to any of the research questions asked. Then, open codes were created while reading the transcripts together (manifest) and organising the data by identifying categories and creating a first set of generic categories (abstraction). In the second round, all preliminary generic categories were discussed together with three other authors (HÅ, GH and BAG), which led to merging, dividing, and re-naming the categories initially developed. Special attention was given to potential overlaps between the generic categories. In the third round, the generic categories were discussed again with one of the authors (HÅ) and all generic categories were checked if they were coherent. All main categories were formulated and visualised through a figure, and a main category was created. Examples of the analytic process from unit of analysis to categories are described in Table 2. Quotations capturing the data were used to illustrate the analytic process. The quotes were translated into English and then re-translated into Swedish to ensure consistency of meanings. We used Excel Office 365 to organise the data during the analysis.

**Table 2:** Examples of the coding trees

Participant group	Units of analysis	Open code	Generic categories	Main categories
PR	You must let go a little of the fact that you’re sitting at a table with both doctors and psychologists and specialist nurses, but still, it comes down to the fact that experience is competence, too	experience is also competence	Informal competence	Finding appropriate patient representativeness
HPR	It is a different competence base; it is an experience-based competence base	experience-based competence		
PR	I joined a Facebook group with 3500 users, partly those who have XXX plus another group those who have XXX … it is a very large group and then I am part of the XXX association, much less activity and information between the members of the national association … there I asked questions and interviewed a few people and got good answers	ask questions and conduct interviews, participate in forums	How to represent	Working methods that facilitate a patient perspective
HPR	a very matter-of-fact way as someone who represents the whole group, she doesn’t just say that “I think this is good” but “We as patients think this is good”. It also signals that she knows others who have been in the same situation, so that she is in some way perceived by the group as actually representing the entire patient group, not just herself.	represent the whole group and not just themselves		
PR	I think people listened to me when I conveyed difficulties … and it emerged in a good way over the course of care itself	they listened to me	Influence	Influence of the patient perspective in the clinical pathways
HPR	It has been very important to have him there and … yes, he offers very good viewpoints and can have a different picture than the other members have, who are mainly healthcare staff then, so it is very important to involve him	good viewpoints, different picture		

PR = patient representative, HPR = healthcare professional representative

## Results

This study identified three main categories in which expressions from both patient representatives and healthcare professional representatives were included. There was a conformity in the experiences shared, with a difference being that patient representatives voiced greater concern regarding the implementation and utilization of the clinical pathways.

**Fig. 2:**
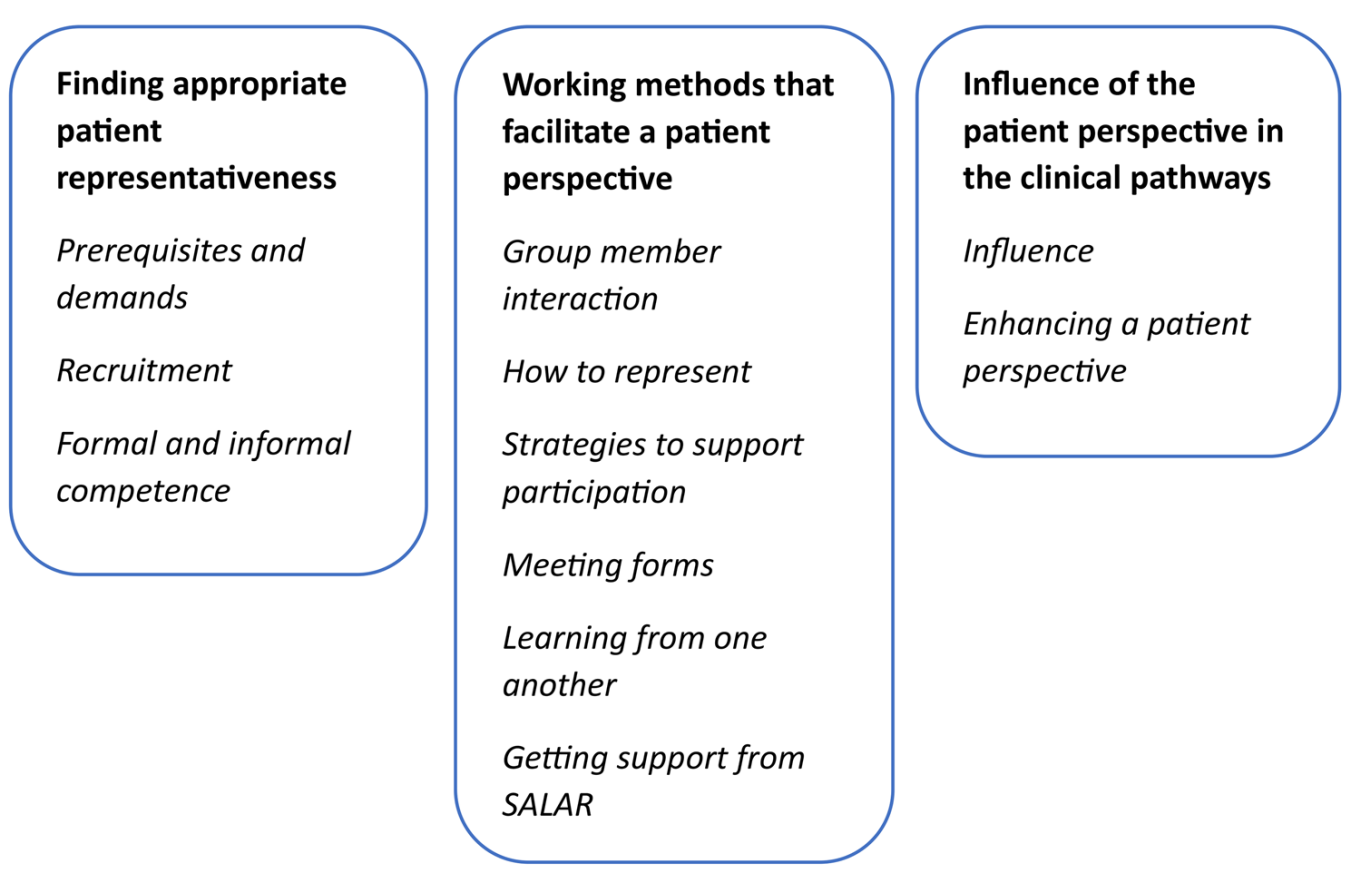
The three main categories and the generic categories

The main categories were: (1) finding appropriate patient representativeness; (2) working methods that facilitate a patient perspective; and (3) influence of the patient perspective in the clinical pathways. The main categories prepared the way towards person-centeredness in the clinical pathways processes (see Fig. 2). In the following we present the main and generic categories, illustrated by quotations from the informants.

### Finding appropriate patient representativeness

We identified a need to clarify *prerequisites and demands* in *recruitment* as well as *formal and informal competence* to find appropriate patient representativeness.

The prerequisites and demands for the recruitment of a patient representative to a national working group were unclear and not explicit. A description of the role was lacking, leading to a feeling of initially working and performing ad hoc (PR5). Not having prerequisites and demands would be equivalent to a risk of “positive special treatment”, an undemanding attitude towards the patient representatives (HPR1). Therefore, the role needed to be clarified, describing the responsibilities of patient representatives and what they were expected to do (HPR8). The remuneration offered for the assignment ought to give room for demands to be made concerning attendance and performance in the working groups (HPR1). Comparisons were made to the specified requirements that existed in the recruitment of other members: “Because we still want this [patient participation] as one of all the others … that’s where we want to get to” (HPR1).

The importance of recruiting appropriate patient representatives for patient participation in the national working groups was stressed. Recruiting from a national patient association provided uniformity in how all members are recruited as “Others in national working groups representing an organisation” (HPR1, HPR4). Recruiting patient representatives could, however, be problematic, as “… we had a hard time finding any [patient representatives] so we had to hunt a bit …” (HPR3). This could be because there was no relevant patient association for the current working group (HPR1) or because some patient groups had not formed a patient organisation, for example when having a certain disease could be experienced as stigmatising and not something you want to show off (HPR4). Recruitment could be facilitated, for example, by having more than one patient representative in a group (HPR4). Being more than one appointed representative had the advantage of being able to cover for each other. Closer contact, dialogue and collaboration with patient associations could also be useful.

Patient participation in a national working group was rather complex, demanding both formal and informal competence. Formal competence was described as patient representatives knowing how to produce material, and how to write and comment on materials that have been produced (HPR2). It was suitable to have experience of working and participating in a group (PR9) and an advantage to have worked with process development (PR3). Formal competence also consisted of being used to interviewing others and forming one’s own opinion (HPR4). Some stressed the need for formal competence of chairpersons and process leaders as well, declaring low competence and inexperience to be a difficulty when working with patient participation (HPR8).

Informal competence was based on patient representatives’ experience, compared to other group members, of being patients in Swedish healthcare and thus having “a different competence base, it is an experience-based competence base …” (HPR1). Patient representatives in the national working groups were “…extremely professional and good at calling in what, how … we can get good answers, or answers that develop care for the target group” (HPR3). Informal competence was also about being receptive, waiting for a good opportunity to describe one’s own experiences. It was “… good to be a little cool in the beginning and not to cling, … to listen …” (PR4), to blend in and be responsive (HPR4). At the beginning of the national working group’s work, it was more uncertain when “… you should go in and explain yourself” (PR1), but as the group work continued it became easier. Informal competence was also about really speaking up when needed (HPR4), listening first and not being shy to give comments and express your opinion later (PR4). Entering a group could be perceived as challenging when working together with highly trained specialists (HPR5). It was therefore important to be confident in oneself, brave and tough, daring to take one’s place (HPR5) and to “… realise that experience is also competence” (PR8). Being a patient representative in a national working group contributed to the development of care, personal development and a greater understanding of the challenges, obstacles and opportunities in health care (PR4).

### Working methods that facilitate a patient perspective

This main category discerned that a patient perspective was facilitated by *group members’ interaction*. It showed the importance of *how one represents*, *strategies to support participation*, *meeting forms*, *learning from each other*, and *getting support from SALAR*.

Group members’ interactions contributed to creating a good climate in the national working groups. There was a friendly tone in the groups, all members had room to speak, patient representatives were listened to and the “personal chemistry was right” (PR3, PR4). In the groups, patient representatives were met by civil discourse and respect (PR1, PR6, PR7, PR9) and felt included (PR1, PR9). Initially some patient representatives experienced feeling inferior in the company of well-educated and well-known professionals: “Of course, you get a little bit star struck when you see the list of participants” (PR4), but rules for group interactions, for example to avoid unnecessary medical language and abbreviations (PR8), led to an experience of feeling equal. Group members being open with their knowledge added to the experience of equality and of “being players at the same level” (PR4). The patient representatives’ interaction in the group was increased by all members having the same focus, namely, to improve healthcare for the patients (PR6). They were more active when the group was divided into smaller groups (PR1, PR8) and when they took part in planning a group meeting (PR8).

Patient participation in the national working groups was multifaceted. The representatives had to represent both their own experience and that of others and have a double perspective: “you don’t represent yourself as a patient or a close relative, of course you contribute with your experiences, that’s how it is, but you also have a task anchor the current work with [those] affected in the network” (HPR7). The double perspective was important to consider when recruiting patient representatives (HPR2, HPR4). Signs of having a double perspective were, for example, saying “we” instead of “I”: “It also signals that she knows others who have been in the same situation, so that in some way she is perceived by the group as actually representing the entire patient group, not just herself” (HPR4). It was also important to have the know-how for which of the two perspectives should be put forward: “Because it is a balancing act right here and the difficulty for the patient representatives to share their own experience but also put a limit where … yes where is the limit for my own … so to speak, commitment, and my own experience linked to the patient group that one represents” (HPR7).

The double perspective was promoted by taking a broader, generalised role, representing all categories of patients, and thinking “… how is it for other patients and not just for me” (PR5). This perspective could be obtained in several ways. Some based the work in the national working group by discussing with the relevant patient organisations (PR1, PR6), with different types of networks (HPR7), Facebook groups (PR5) or interviews with members in relevant patient organisations (PR5). Establishing their own opinion with a broader group as reference point gave a sense of self-assurance (PR8) and made it easier to take responsibility for their contributions to the content in the clinical pathway (PR6).

Patient participation was strengthened by a variety of strategies. Healthcare professional representatives had separate meetings with the patient representatives, digitally, by telephone and email contact, where they offered information and could ask questions and reflect on their experiences of working in the national working group (HPR6). During the group’s working meetings, one strategy was to divide the group into smaller ones. Another was an inviting strategy to include and involve patient representatives. This was done, for example, by directing specific questions to them: “Do you have something to say here? You can also write something in the chat …” (HPR2). The inviting strategy was important for patient representatives to feel welcomed in the group (PR8). This strategy was especially important if the discussion among other members became too medically oriented (HPR2). Another strategy was to help patient representatives not to overemphasise and talk too much about their own experiences (HPR1). If such a situation occurred, they were supported by “leading them [the PRs] away” from their own medical histories and their own diagnoses (HPR2).

The meeting forms in the national working groups varied and were conducted physically and digitally. Physical meetings gave room to get to know one another better compared to meeting via digital platforms. For some, an introductory meeting was held physically, giving them the chance to get to know one another and to ask questions about the assignment (PR6). At a physical meeting, more questions can be asked (PR2). Due to the restrictions surrounding the COVID-19 pandemic, however, the physical meetings switched to digital ones. For some patient representatives, all working group meetings were digital. During digital meetings, they did not want to interrupt and ask, “beginner questions” (PR3). However, digital meetings were time saving as participants in the working groups came from all over the country (PR7). Forming small groups digitally could slightly compensate as a way for group members to get to know each other better and a way for the knowledge and opinions of patient representatives to be expressed in a better way (PR1).

Patient participation in national working groups enhanced learning for both healthcare professional and patient representatives. The latter gained deeper knowledge both on the specific disease and on scientific matters. Participation in the group opened a new world for them, for example by grasping the complexity of healthcare (PR1, PR2, PR3). They also learned from other patient representatives. In working groups with two or more patient representatives, they supported each other, which increased the weight of their arguments and made them stronger (PR6, PR7, PR8). Being a patient representative in a working group together with a more experienced patient representative was experienced as boosting (PR4). They were often interconnected and had the same opinion but could also speak up if they had different thoughts on a specific matter (PR4). Healthcare professional representatives also learned from the patient representatives, for example, “to get feedback and have a dialogue with the patient representatives, what has been good, what has been less good, what can we develop further and do better. I feel that I have learned from the participants” (HPR7).

The SALAR administrative support function’s knowledge on the details in the reimbursement system was considered important (HPR2) and supporting materials, for example films that described what it is like to participate in a national working group, were helpful to provide an understanding of the role as patient representative (HPR2). Supporting materials were used especially when process leaders and chairpersons were new to their roles (HPR2). The support for patient participation could be further improved by involving professional communicators in the process at an early stage (PR7), and by, for example, improving the process of disclosing conflicts of interest (PR5). Patient representatives sought feedback and input from the administrative support function regarding the outcomes of the clinical pathways implementation and how it could improve healthcare overall. (PR7).

### Influence of the patient perspective in the clinical pathways

This main category showed that both working group meetings and the clinical pathways were *influenced* by the input from the patient representatives and that patient participation contributed to *enhancing a patient perspective*.

Patient representatives thought that their experiences and views received attention and were integrated into the work, and that they had contributed to the clinical pathways both in the working process and in the final text (PR4). The ability to influence the clinical pathways and hence contribute to equal care was considered important (PR1). Patient representatives had also led to changes in decisions on the scope of clinical pathways (HPR4) and to changes in the title of the clinical pathways.

The ability to influence was initially limited in the working process but increased with time (PR1). The patient representatives’ statements and utterances sometimes had a strong and convincing effect: “When they say something, the group stops. It’s not that a nurse or a doctor doesn’t know what civil discourse is. But when a patient representative says it, it becomes truth in a different way” (HPR1). The patient representatives’ utterances were more influential, compared to the professionals’ (HPR8). Patient experiences worked as something of an eye-opener; what was obvious to the patients was not so obvious to the healthcare professionals (HPR4, HPR5). Patient representatives came up with aspects that the professionals never had considered or even thought of (PR7). Besides participating in national working groups, patient representatives contributed to national information meetings describing the clinical pathways (HPR7), but worried about the readiness for implementation in the regions (PR1). They were surprised that healthcare was performed differently in different parts of the country and therefore considered it valuable to have national clinical pathways covering all Swedish regions (PR4). However, patient representatives feared that the work invested would be wasted and that the clinical pathways would become another piece of paper that was not used or did not result in equal care (PR6).

Patient participation in the national working groups gave healthcare professional representatives a deeper understanding of the patient representatives’ work in developing clinical pathways. Their participation had worked well and including them in the working group was seen as an expression of person-centeredness. Patient representatives were considered brave, bringing forward their experiences in such a way that other participants in the working groups were impressed (HPR5). The value of patient representatives participating in producing clinical pathways was evident as these were intended as a knowledge base for healthcare meetings, in which the patient is a partner (HPR3, HPR7): “You cannot have a national working group without patient representatives, in the same way that you cannot work with person-centeredness without participation of the people to whom the clinical pathway is addressed” (HPR2). Patient participation in the national working groups contributed to healthcare personnel moving from an organisational perspective to a patient perspective (HPR8).

## Discussion

This study of patient participation when co-producing person-centred and cohesive clinical pathways at the national level contributes to the growing body of literature studying patient participation [17, 30]. We identified three main categories which add to our knowledge of what to expect and consider when using patient representatives in the development of clinical pathways.

One main category shows the importance of a careful recruitment process, demanding formal requirements and informal competence of the potential patient representatives. Notably, both patient and healthcare professional representatives emphasise the importance of a clear structure for reimbursement and requirement profile for patient representatives in the national working groups. This study demonstrates the unique competence of the patient representatives. The competence to represent a dual perspective – to speak for oneself and for others – places high demands on patient representatives at the national level, which also applies to all participants in the national working group. The need for a dual perspective could benefit from being made more explicit to all participants.

The second main category shows the importance of healthcare professional representatives’ strategies for strengthening patient participation. Before and during working group meetings, they can support patient representatives in dealing with the dual perspective and balancing when one or the other perspective should be in the foreground. It is likely that their efforts to achieve a sound balance and interaction within the groups mattered. Another strategy to strengthen patient participation might also be to offer them support through the exchange of experiences with other patient representatives, for example in network meetings and through mentorship from more experienced patient representatives. It is also interesting that several of the patient representatives emphasised the advantage of being at least two in a group, indicating that they might experience subordination, perhaps a feeling of imbalance in the number of representatives and maybe also in knowledge.

The learning that takes place within the working groups is important. The organisational support for patient participation at SALAR was not developed in detail when the system first started but has evolved and improved over time. Over the years, routines for how to work with patient participation have been developed, built on the experiences of the national working groups, which strengthens the point that the system works as a “learning health organisation”. According to Elwyn et al. [1], there is a connection between co-production with the voice of the patient and practice improvement and organisational design. The idea of co-production could contribute to the idea of a learning health system [6], which is a goal of the national system for knowledge-based management.

The third main category shows that patient representatives’ experiences are expressed in the finalised clinical pathways. Their contribution to the mandatory patient pathway was necessary to achieve credibility. It also shows that it is vital to include patient participation in the national working groups to promote person-centeredness. As mentioned before, enhancing person-centred care is a goal of the national system. However, person-centred care is a debated concept that has been described as vague, multi-faceted and not well-defined, lacking a precise definition [31]. Person-centred care can be narrowed down to three aspects: (a) understanding patients’ experience of illness and their life situation, (b) the professional’s relationship with the patient, and (c) the coordination of care. An updated “definition of patient-centred care and its operationalization can make its implementation in healthcare more manageable” [31]. Patient participation in national working groups contributes to these aspects by integrating the unique knowledge of patient representatives’ lived experience, the relationships with the professionals and the encounters with the total healthcare system into the clinical pathways. With co-production, the risks of fragmented healthcare and barriers to accessing healthcare services can be identified, and awareness of the pitfalls in coordinated care can increase [18].

The study shows that the system is developing when it comes to patient participation. This study contributes to further developing how patient participation can be enhanced. It can add to the adoption of the principles of co-production in healthcare, which is still slow [1]. We also see the possibility for the system to take advantage of the results of patient participation on all levels of healthcare, in accordance with Carman et al., on micro, meso and macro levels [15].

### Methodological considerations

A strength of this study is that both patient and healthcare professional representatives acting at a national level were interviewed, which seems quite rare in the literature [28]. The study uses an interactive approach where the process of joint learning is central [33]. Patient representatives included in the research group took part in every step of the research process throughout the project. Their input led to revisions of the semi-structured interview guides, thereby increasing the relevance of the questions asked. The feasibility of the data collection methods was discussed and enhanced jointly, and the patient representatives checked the coding and took part in grouping the categories under higher order headings. Working interactively contributed to learning more about patient participation throughout the process.

Using qualitative methods allowed for asking the informants probing questions that would not have been possible in questionnaires. We were able to include informants strategically, as intended, with a broad variety of informants working in a wide range of national working groups to reflect the system. This should strengthen the transferability of the results. We initially planned to include approximately ten patient and ten healthcare professional representatives, considering the extent, specificity and theoretical connection of the aim and previous experience of the research team. Theoretical saturation was reached after having included nine patient representatives and eight healthcare professional representatives, respectively. A limitation of the study is that only chairpersons and process leaders in national working groups were interviewed. Hence, an area for further research is to also include other group members.

Individual interviews allowed the informants to freely express their experiences. The interviews were led by two researchers (CP or SM). One researcher conducted. the interview, while the other researcher ensured that all questions outlined in the interview guide was covered and provided prompting questions to help clarify the participant’s descriptions [32].

An inductive approach to analysing the data was found appropriate since patient participation at system level is less explored. Digital interviews may be seen as a drawback. However, our experience as researchers and reviewers is that digital contacts have been more common since the start of the COVID-19 pandemic. Digital recordings enabled the third researcher involved in the analyses not only to listen to audio recordings and read transcripts, but also to observe mimics and body language. One of the researchers had a deeper pre-understanding of the informants due to her previous work at the national administrative support function. There is always a risk that this would cause the informants to be more cautious about expressing criticism in their feedback, however nothing in the interviews indicated that this was the case. The pre-understanding of the system can also add to a deeper understanding. Credibility further increased by having all members of the research group, including patient representatives, checking, and discussing all steps in the analysis. The link between the data and the analytical steps with authentic quotations also supported the findings to increase the reliability of the interpretation of the data.

## Conclusion

This study shows the importance of patient representatives’ participation in the co-production of clinical pathways. Patient participation takes time to develop into co-production. To achieve an impact, a thoughtful recruitment process considering the dual perspective, that is, to represent oneself and others, should be considered. Likewise, actions to strengthen the interactions in the working groups can help the patient representatives’ competence to come to the fore.

Experiences of patient participation in the development of clinical pathways on the national level can also be helpful when the clinical pathways are implemented in the actual care, that is, at the micro level. The findings in this study provide a platform for further research, for example for observational studies of interactions in the working groups as well as studies on the interplay between the different levels of the care system when the guidelines for clinical pathways are to be used systematically at the local level.

In this study, patient representatives took part in the planning, analysis, and the final version of publication phase of the research project. This was important as the initial research questions were refined and developed, and the categories were constructed jointly.

### Key learnings and practical implications

- Clarifying requirements regarding patient representatives’ formal and informal competence may facilitate the recruitment process.
- There is room for improvement of methods and tools to enhance patient representatives’ competence to represent others.
- Patient representatives’ participation is supported by initial physical meetings.
- Representation from a patient organisation enables broad perspectives for patient representatives’ participation.
- More than one patient representative is recommended in each group.
- The strategies and competence of process leaders and chairpersons to lead a group are important to facilitate patient participation.
- Patient participation is enhanced by administrative support to coordinate patient representative meetings for learning and exchanging experiences.

### Electronic supplementary material

Below is the link to the electronic supplementary material.

Supplementary Material 1

Supplementary Material 2

## Data Availability

No datasets were generated or analysed during the current study.

## Abbreviations

PR: patient representative
HPR: healthcare professional representative
SALAR: The Swedish Association of Local Authorities and Regions
