# Supplementary material for: Experiences of co-producing person-centred and cohesive clinical pathways in the national system for knowledge-based management in Swedish healthcare: a qualitative study
Source: Res Involv Engagem. 2024 Jun 7;10:55. doi: 10.1186/s40900-024-00565-3 (PMC11157721; doi:10.1186/s40900-024-00565-3)
Supplement: Supplementary file 1 — Supplementary Material 1 [file 40900_2024_565_MOESM1_ESM.docx]

**Additional File 1: GRIPP2 Short Form Checklist**

| **Section and topic** | **Item** | **Reported on page No** |
| --- | --- | --- |
| 1. Aim | Study aim was to explore experiences of patient participation from the perspectives of both patient and healthcare professional representatives when co-producing clinical pathways within the nationwide system for knowledge-based management of Swedish healthcare. Does the participation influence the end-products | 8 |
| 2. Methods | Interview guides were developed together with two patient representatives. Discussions during the analytic process was made with two patient representatives. | 12 |
| 3. Study results | We identified three main categories and a supportive base, which together add to our knowledge of what to expect and consider when using patient representatives in the development of clinical pathways. | 14-24 |
| 4. Discussion and conclusions | In this study, patient representatives took part in the planning, analysis and the final version of publication phase of the research project. This was important as the initial research questions were refined and developed, and the categories were constructed jointly. | 24-29 |
| 5. Reflections/critical perspective | This study shows the importance of patient representatives’ participation in the co-production of clinical pathways. Patient participation takes time to develop into co-production. | 29 |
