# Supplementary material for: Experiences of co-producing person-centred and cohesive clinical pathways in the national system for knowledge-based management in Swedish healthcare: a qualitative study
Source: Res Involv Engagem. 2024 Jun 7;10:55. doi: 10.1186/s40900-024-00565-3 (PMC11157721; doi:10.1186/s40900-024-00565-3)
Supplement: Supplementary file 2 — Supplementary Material 2 [file 40900_2024_565_MOESM2_ESM.docx]

**Additional file 2 Question guides**

*Question guide I*

Patient and next-of-kin representatives in the National system for knowledge-based management – Sweden’s regions in collaboration

• Which national working groups do you participate in?

• How many patient representatives are there in the working groups in which you participate?

• How were you recruited?

• How was your introduction? How were you introduced to the working group?

• There is an agreement to clarify the assignment when participating in working groups. Have you used it?

• What did you think of the introduction; did it provide opportunities for discussion, reflection and consensus regarding the mission and purpose of the group/programme area?

• How clearly is the task of the patient/next-of-kin representative described?

• How do you feel about your role as patient/next-of-kin representative in the working group?

• What do you think are the conditions for participating in the working groups?

• How do you think your views, experiences and perceptions are taken care of?

• Participation can take different forms. How would you describe how it has been for you in this group (e.g. from 1–6, where 1 is the least participation to 6 which is the highest participation)? (Information, consultation, dialogue, influence, co-decision-maker)

• What is your perception of your meetings: have they been digital, have you met physically and has there been any difference?

• Would you recommend someone else to be a patient/next-of-kin representative?

• Do you have any advice for new patient representatives who are just about to start their assignment?

• Is there anything else you would like to add?

*Question guide II*

Process leaders, chairpersons of the National system for knowledge-based management – Sweden’s regions in collaboration

• What role do you have in the knowledge management system?

• Are there any appointed patient/next-of-kin representatives in your national working groups?

• How has the work with patient/next-of-kin representatives worked as a whole?

• How were patient/next-of-kin representatives recruited?

• How have patient/next-of-kin representatives been introduced?

• Did the introduction entail opportunities for discussion, reflection and consensus regarding the group’s aim and purpose?

• Have you used the routine for patient participation in your work with patient participation? If so, how?

• Can you describe how you experience having patient/next-of-kin representatives involved in the work?

• How clearly are the assignments for patient/next-of-kin representatives described?

• How do you experience the role of patient/next-of-kin representative in the working group?

• What are the conditions for the patient/next-of-kin representatives to participate in the working groups, do you think?

• How do you think the views, experiences and perceptions of the patient/next-of-kin representatives are taken into account?

• Can you give concrete examples of the patient/next-of-kin representatives’ contribution?

• Is there anything else you would like to add?
